# Supplementary material for: Analysis of policy interventions to attract and retain nurse midwives in rural areas of Malawi: A discrete choice experiment
Source: PLoS One. 2021 Jun 21;16(6):e0253518. doi: 10.1371/journal.pone.0253518 (PMC8216531; doi:10.1371/journal.pone.0253518)
Supplement: S1 File — (DOCX) [file pone.0253518.s001.docx]

**S1 File. Qualitative Interview and Focus Group Discussion Guides**

1. Qualitative interview guide for deployed nurses

This interview guide is meant to be used to explore health workers’ motivations for working in rural areas. The interview guide should only be employed after the participant has provided informed consent. If participants seem hesitant to respond to any questions, you can remind them that their answers are confidential, but also that they do not have to respond if they do not want to.

Ice breaker questions:

1. Tell me how you got yourself into the nursing profession?
   1. Where were you trained?
   2. What or who inspired you to become a nurse?
2. Tell me about your family?
   1. Are you married? How big is your family? Do you have children? How many?
3. What are you most proud of in life? Why?

General experiences and satisfaction with the nursing profession:

1. Why did you choose to become a nurse? What are the things that motivated you become a nurse?
   1. How important are the things that made you choose to be a nurse, to you today?
   2. How similar is your current position to the way that you expected it to be?
2. How long have you worked at this health facility?
   1. Have you worked at other health facilities prior to working at this facility?
   2. Have you ever imagined being transferred to other facilities? Where? Why?
3. What do you like about your job?
   1. Do you think that other nurses enjoy similar things about their jobs? Why?
4. Are there aspects of your job you wish could change?
   1. What are these aspects?
   2. What would it take to make that change happen? Who would have to be responsible for it? Do you think it is possible? How?

Job preferences and work location:

1. This country has a wide range of type of communities, from urban to very rural. What type of community would you most like to work in?
   1. Why do you think you prefer that kind of area?
   2. Would you be happy in any type of (urban/rural) area, or are there certain community characteristics or parts of the country that would be better for you?
   3. What are the benefits to working in an urban area?
   4. What are the challenges of working in an urban area?
   5. What are the benefits of working in a rural area?
   6. What are the challenges of working in a rural area?

Job attributes:

1. What are some of the important issues you consider when thinking about where you want to work?

*[Note: Allow the participant to mention the issues that come to mind, and probe to better understand those issues. After they finish, you can ask about the following issues if they were not already mentioned:]*

- 1. Scope of Work: Do you think working in rural areas has an impact on the kind of health care you would prefer to provide or on your scope of practice? How important is this to you when deciding where you will work?
  2. Quality of facilities and equipment: Do you think that health facilities in rural areas are of different quality than facilities in urban areas? How important is the quality of the facility to you when deciding where you will work? What particular factors are most important, i.e. high-tech equipment, constant drug supply, etc.?
  3. Management structure: Do you think that there is anything different about the management structure of facilities in rural areas? Are managers in rural areas more difficult or easier to work with than those in urban areas? How important is management to you when deciding where you will work?
  4. Distance to a city: Is the distance a rural posting is from a bigger urban city important to you when deciding where you will work? How far away from an urban city would you be willing to work?
  5. Distance to family: Is the distance a rural posting is from your own family important? How far away from your family would you be willing to work?
  6. Living conditions: What is your opinion of the living conditions in rural areas? How important are the living conditions for you and your family when considering where you will work? What aspects of living conditions are more important (housing, schools, etc.)?
  7. Career Advancement: Do you think that working in a rural area would have an impact on your ability to advance your career? How important are opportunities for career advancement to you when considering where you will work?
  8. Bonding arrangements: Do you think that working in the rural area with a different bonding arrangement would have an impact on your willingness to stay in the rural areas.
  9. Rotation arrangements: If there was an arrangement to rotate nurses between urban and rural health facilities and vice versa:
     - Would you be willing to work in the rural area knowing someday you will move to an urban facility? How long would you want to work in a rural health facility before you are moved to urban health facility?
     - Would you be willing to work in urban health facilities knowing that someday you will be transferred to a rural facility? How long would you want to work in an urban health facility before you are moved to a rural health facility?

Conclusion

1. What do you think is the most important change that could occur in the job conditions of nurses and midwives in Malawi to increase satisfaction and retention in the rural areas?
   1. How important is that change in making decisions for most nurses to work and stay in rural areas?
   2. Why is this change such a high priority for nurses like yourself?
2. What would you need to make your work environment and living conditions better/more livable? Please put these attributes in order of priority.

| Housing |  |
| --- | --- |
| Facility Level Infrastructure |  |
| Location – Rural/Urban |  |
| Proximity to Home District/Family |  |
| Professional Environment/Supportive Management |  |
| Ability to Rotate within Bonding Period |  |
| Workload |  |
| Transport |  |
| Community and Broader Network of Support |  |
| Bonding Period |  |
| Rotation |  |
| Career Advancement |  |

1. If Malawi created a package of benefits to motivate nurses in rural areas, what are the top three priorities (not including salary increases, bonuses or monetary benefit) that would be the most important to include?
   1. Why would you include that?
   2. What are some of the concerns you may have around any of these attributes?
2. **Focus group discussion guide for graduating nurses**

This focus group discussion guide is meant to be used to explore health workers’ motivations for working in rural areas. The focus group discussion guide should only be employed after the participant has provided informed consent. If participants seem hesitant to respond to any questions, you can remind them that their answers are confidential, but also that they do not have to respond if they do not want to.

Ice breaker questions:

1. In order to get started, I’d like to learn a little bit more about your background. So first, I’d like everyone to go around and tell me a little bit about yourself:
   1. What you were doing before you started this program?
   2. Where did you grow up? Which district? Was it in a town or in a rural area?
   3. In nursing, who is your model and why do you look up to them as models?
2. How do you start your routine day when you are doing course work on campus?

Experience working and living in rural areas:

1. Why did you choose to become a nurse?
   1. What are your expectations after completion of your nursing course?
   2. What are you most proud of now as you prepare to join the nursing profession?
   3. What are your fears now as you prepare to join the nursing profession?
2. Have you ever experienced working (in a nursing capacity) in a rural area (such as during clinical rotations)?
   1. What did you like about it?
   2. What did you dislike about it?
3. Describe a health facility in your village?
   1. Do you think it provides the best care to patients? Why?
   2. Do you think health workers are happy providing their services there? Why?
   3. If you were deployed to work in a health center in your village/near your village how would you have loved it to be improved to make it better for your stay? What are some of the things you would consider? Why?
4. What is your definition of a rural health facility?
   1. What are some of the characteristics you’re likely to see or associate with a rural health facility?

Main questions:

1. What are some of the important issues you consider when thinking about a health facility where you want to work?
   1. Would you be happy in any type of (urban/rural) area, or are there certain community characteristics or parts of the country that would be better for you?
2. If you were made to choose your ideal placement for after graduation, where would it be?
   1. Ask about details, district (why chosen)?
   2. Urban vs. rural (why chosen)?
   3. Type of facility, such as rural health center vs. urban health facility (why chosen)?
   4. Closer to your village vs. far from your village (why chosen)?
3. What are some of the challenges you or your family might face when posted at rural health facility?
   1. Which ones are most important and why?
   2. How important are these challenges to you and your family when deciding where you will work? Why?
4. What could motivate you to work in the rural health facility? Why? (i.e., high-tech equipment, constant drug supply, housing, transportation etc.)
   1. How important are these things you mentioned in deciding where you will work? Why?
5. If you were in a position of authority, how would you make rural health facilities the best place to work in?
   1. What other general crosscutting characteristics would you consider to improve in rural health facilities?
6. What are some of the challenges you or your family might face when posted at an urban health facility such as a District or Community Hospital?
   1. Which ones are most important challenges and why?
   2. How important are these challenges to you and your family when deciding where you will work? Why?
7. What could motivate you to work in an urban health facility? Why? (i.e., high-tech equipment, constant drug supply, housing, transportation etc.)
   1. How important are these things you mentioned in deciding where you will work? Why?
8. If you were in a position of authority, how would you make urban health facilities the best place to work in?
   1. What other general crosscutting characteristics would you consider to improve in urban health facilities?
9. What are the best ways to continue to grow in your profession? (i.e., improving your professional skills)
   1. What support would you require to progress?
   2. How important are opportunities to grow in deciding where you want to work?
10. How do you think working in a rural area will affect your career advancement?
    1. How important are opportunities for career advancement to you when considering where you will work?
11. Based on your visit to rural health centers during your clinical rotations, what do you think your experience would be if you imagined working there full-time?
    1. Which part would be interesting? Why?
    2. Which parts in particular do you feel would be the most challenging? Why?
12. Are there any other factors that are important to you when deciding where you will work?
    1. Is the distance a rural posting is from a bigger urban city important to you when deciding where you will work?
    2. How far away from an urban city would you be willing to work?
    3. Assuming working in the rural and urban would have different bonding lengths, would bonding be an important factor to decide where you would be willing to work?
    4. How long would an ideal bonding time period be for working in a rural area? Why?
13. Which types of benefits or incentives would be most important for you in deciding to work in a rural area?

*[Note: The interviewer may provide some examples of incentives if necessary, but first let the participant suggest incentives that they can think of without any prompting.]*

- 1. What advice would you give to policy makers who are considering different types of incentives to increase staffing at rural health facilities?

1. If there was an arrangement to rotate nurses between urban and rural health facilities and vice versa:
   1. Would you be willing to work in the rural area knowing someday you will move to an urban facility? How long would you want to work in a rural health facility before you are moved to an urban health facility?
   2. Would you be willing to work in urban health facilities knowing that someday you would be transferred to rural facility? How long would you want to work in an urban health facility before you were moved to a rural health facility?
2. Where do you see yourself in the next 5 years?
   1. What do you need to achieve that vision?
   2. How would the things you listed be important to you when considering where you will work?
3. Where do you see yourself in the next 10 years?
   1. What do you need to achieve that vision?
   2. How would the things you listed be important to you when considering where you will work?

Group ranking activity:

1. We have now discussed several unique factors that may affect your decision on where you choose to work as a health professional. These factors include:

*[Note: The FGD leader should have a flip chart at all times, and each time a new issue comes up, they should write it on the board throughout the conversation. Then at the end, they can just refer to that list, which might include the following areas.]*

- 1. Housing provision or support at place of work
  2. The quality of facilities, including availability of technology, drug supply, etc.
  3. The management environment at facilities; sense of community and mentorship at the workplace
  4. Distance from urban city; distance from home district (if placed outside of home district)
  5. Living conditions for you and your family at the place of your work
  6. Opportunities for career advancement
  7. Transport to and from place of work / transport easily available
  8. Level of workload
  9. Bonding

1. Thinking about these factors, please pick out the four most important and rank them in order starting with the most important:
   1. Why have you arranged these factors in that order?
   2. If you were to think of another important factor not on this list, what would it be and how would you rank it among the four and why?
