# Supplementary material for: Analysis of policy interventions to attract and retain nurse midwives in rural areas of Malawi: A discrete choice experiment
Source: PLoS One. 2021 Jun 21;16(6):e0253518. doi: 10.1371/journal.pone.0253518 (PMC8216531; doi:10.1371/journal.pone.0253518)
Supplement: S2 File — (DOCX) [file pone.0253518.s002.docx]

**S2 File. Twelve Choice Sets in Block A of the Discrete Choice Experiment Questionnaire**

|  | **Rural Facility** | **Urban Facility** |
| --- | --- | --- |
| Housing | Free superior housing provided (eg. detached house with reliable electricity and three bedrooms) | No housing or housing allowance provided |
| Health Facility Quality | Advanced (e.g. reliable electricity, equipment and drugs and supplies always available) | Advanced (e.g. reliable electricity, equipment and drugs and supplies always available) |
| Access to Upgrading | Eligible to apply for upgrading opportunities after 4 years of service | Eligible to apply for upgrading opportunities after 4 years of service |
| Workload | Heavy workload - You work longer hours because the facility does not have enough staff | Heavy workload - You work longer hours because the facility does not have enough staff |
| Facility Management | The management at the facility is not supportive and makes work more difficult | The management at the facility is supportive and makes work easier |
| Salary | 187,604 MKW per month | 187,604 MKW per month |
| Choice of Location | You are given a choice of district in which you will work | You are randomly assigned to a health facility |
| **Which job do you choose?** |  |  |

|  | **Rural Facility** | **Urban Facility** |
| --- | --- | --- |
| Housing | Free superior housing provided (eg. detached house with reliable electricity and three bedrooms) | No housing or housing allowance provided |
| Health Facility Quality | Basic (e.g. unreliable electricity, equipment and drugs and supplies not always available) | Advanced (e.g. reliable electricity, equipment and drugs and supplies always available) |
| Access to Upgrading | Eligible to apply for upgrading opportunities after 3 years of service | Eligible to apply for upgrading opportunities after 3 years of service |
| Workload | Manageable workload - You work within scheduled hours because the facility has sufficient staff | Heavy workload - You work longer hours because the facility does not have enough staff |
| Facility Management | The management at the facility is supportive and makes work easier | The management at the facility is not supportive and makes work more difficult |
| Salary | 156,365 MKW per month | 125,069 MKW per month |
| Choice of location | You are randomly assigned to a health facility | You are randomly assigned to a health facility |
| **Which job do you choose?** |  |  |

|  | **Rural Facility** | **Urban Facility** |
| --- | --- | --- |
| Housing | Free superior housing provided (eg. detached house with reliable electricity and three bedrooms) | No housing or housing allowance provided |
| Health Facility Quality | Basic (e.g. unreliable electricity, equipment and drugs and supplies not always available) | Advanced (e.g. reliable electricity, equipment and drugs and supplies always available) |
| Access to Upgrading | Eligible to apply for upgrading opportunities after 3 years of service | Eligible to apply for upgrading opportunities after 4 years of service |
| Workload | Heavy workload - You work longer hours because the facility does not have enough staff | Manageable workload - You work within scheduled hours because the facility has sufficient staff |
| Facility Management | The management at the facility is not supportive and makes work more difficult | The management at the facility is not supportive and makes work more difficult |
| Salary | 125,069 MKW per month | 156,365 MKW per month |
| Choice of location | You are given a choice of district in which you will work | You are given a choice of district in which you will work |
| **Which job do you choose?** |  |  |

|  | **Rural Facility** | **Urban Facility** |
| --- | --- | --- |
| Housing | No housing or housing allowance provided | No housing or housing allowance provided |
| Health Facility Quality | Advanced (e.g. reliable electricity, equipment and drugs and supplies always available) | Basic (e.g. unreliable electricity, equipment and drugs and supplies not always available) |
| Access to Upgrading | Eligible to apply for upgrading opportunities after 3 years of service | Eligible to apply for upgrading opportunities after 4 years of service |
| Workload | Heavy workload - You work longer hours because the facility does not have enough staff | Heavy workload - You work longer hours because the facility does not have enough staff |
| Facility Management | The management at the facility is supportive and makes work easier | The management at the facility is supportive and makes work easier |
| Salary | 156,365 MKW per month | 187,604 MKW per month |
| Choice of Location | You are given a choice of district in which you will work | You are given a choice of district in which you will work |
| **Which job do you choose?** |  |  |

|  | **Rural Facility** | **Urban Facility** |
| --- | --- | --- |
| Housing | No housing or housing allowance provided | No housing or housing allowance provided |
| Health Facility Quality | Advanced (e.g. reliable electricity, equipment and drugs and supplies always available) | Basic (e.g. unreliable electricity, equipment and drugs and supplies not always available) |
| Access to Upgrading | Eligible to apply for upgrading opportunities after 3 years of service | Eligible to apply for upgrading opportunities after 3 years of service |
| Workload | Heavy workload - You work longer hours because the facility does not have enough staff | Heavy workload - You work longer hours because the facility does not have enough staff |
| Facility Management | The management at the facility is not supportive and makes work more difficult | The management at the facility is not supportive and makes work more difficult |
| Salary | 125,069 MKW per month | 125,069 MKW per month |
| Choice of location | You are randomly assigned to a health facility | You are randomly assigned to a health facility |
| **Which job do you choose?** |  |  |

|  | **Rural Facility** | **Urban Facility** |
| --- | --- | --- |
| Housing | No housing or housing allowance provided | No housing or housing allowance provided |
| Health Facility Quality | Basic (e.g. unreliable electricity, equipment and drugs and supplies not always available) | Advanced (e.g. reliable electricity, equipment and drugs and supplies always available) |
| Access to Upgrading | Eligible to apply for upgrading opportunities after 4 years of service | Eligible to apply for upgrading opportunities after 4 years of service |
| Workload | Manageable workload - You work within scheduled hours because the facility has sufficient staff | Manageable workload - You work within scheduled hours because the facility has sufficient staff |
| Facility Management | The management at the facility is supportive and makes work easier | The management at the facility is supportive and makes work easier |
| Salary | 125,069 MKW per month | 125,069 MKW per month |
| Choice of location | You are given a choice of district in which you will work | You are randomly assigned to a health facility |
| **Which job do you choose?** |  |  |

|  | **Rural Facility** | | **Urban Facility** |
| --- | --- | --- | --- |
| Housing | Free Basic housing provided (eg. semi-detached house with two bedrooms) | No housing or housing allowance provided or housing allowance provided | |
| Health Facility Quality | Basic (e.g. unreliable electricity, equipment and drugs and supplies not always available) | Advanced (e.g. reliable electricity, equipment and drugs and supplies always available) | |
| Access to Upgrading | Eligible to apply for upgrading opportunities after 3 years of service | Eligible to apply for upgrading opportunities after 4 years of service | |
| Workload | Manageable workload - You work within scheduled hours because the facility has sufficient staff | Heavy workload - You work longer hours because the facility does not have enough staff | |
| Facility Management | The management at the facility is not supportive and makes work more difficult | The management at the facility is not supportive and makes work more difficult | |
| Salary | 187,604 MKW per month | 125,069 MKW per month | |
| Choice of Location | You are randomly assigned to a health facility | You are randomly assigned to a health facility | |
| **Which job do you choose?** |  |  | |

|  | **Rural Facility** | **Urban Facility** | |
| --- | --- | --- | --- |
| Housing | Free superior housing provided (eg. detached house with reliable electricity and three bedrooms) | | No housing or housing allowance provided |
| Health Facility Quality | Advanced (e.g. reliable electricity, equipment and drugs and supplies always available) | | Advanced (e.g. reliable electricity, equipment and drugs and supplies always available) |
| Access to Upgrading | Eligible to apply for upgrading opportunities after 4 years of service | | Eligible to apply for upgrading opportunities after 3 years of service |
| Workload | Manageable workload - You work within scheduled hours because the facility has sufficient staff | | Heavy workload - You work longer hours because the facility does not have enough staff |
| Facility Management | The management at the facility is supportive and makes work easier | | The management at the facility is supportive and makes work easier |
| Salary | 125,069 MKW per month | | 156,365 MKW per month |
| Choice of Location | You are randomly assigned to a health facility | | You are given a choice of district in which you will work |
| **Which job do you choose?** |  | |  |

|  | **Rural Facility** | **Urban Facility** |
| --- | --- | --- |
| Housing | No housing or housing allowance provided | No housing or housing allowance provided |
| Health Facility Quality | Advanced (e.g. reliable electricity, equipment and drugs and supplies always available) | Advanced (e.g. reliable electricity, equipment and drugs and supplies always available) |
| Access to Upgrading | Eligible to apply for upgrading opportunities after 4 years of service | Eligible to apply for upgrading opportunities after 4 years of service |
| Workload | Manageable workload - You work within scheduled hours because the facility has sufficient staff | Manageable workload - You work within scheduled hours because the facility has sufficient staff |
| Facility Management | The management at the facility is not supportive and makes work more difficult | The management at the facility is not supportive and makes work more difficult |
| Salary | 187,604 MKW per month | 156,365 MKW per month |
| Choice of Location | You are randomly assigned to a health facility | You are given a choice of district in which you will work |
| **Which job do you choose?** |  |  |

|  | **Rural Facility** | **Urban Facility** |
| --- | --- | --- |
| Housing | No housing or housing allowance provided | No housing or housing allowance provided |
| Health Facility Quality | Basic (e.g. unreliable electricity, equipment and drugs and supplies not always available) | Basic (e.g. unreliable electricity, equipment and drugs and supplies not always available) |
| Access to Upgrading | Eligible to apply for upgrading opportunities after 4 years of service | Eligible to apply for upgrading opportunities after 3 years of service |
| Workload | Manageable workload - You work within scheduled hours because the facility has sufficient staff | Manageable workload - You work within scheduled hours because the facility has sufficient staff |
| Facility Management | The management at the facility is not supportive and makes work more difficult | The management at the facility is not supportive and makes work more difficult |
| Salary | 156,365 MKW per month | 187,604 MKW per month |
| Choice of Location | You are randomly assigned to a health facility | You are randomly assigned to a health facility |
| **Which job do you choose?** |  |  |

|  | **Rural Facility** | **Urban Facility** |
| --- | --- | --- |
| Housing | Free Basic housing provided (eg. semi-detached house with two bedrooms) | No housing or housing allowance provided |
| Health Facility Quality | Basic (e.g. unreliable electricity, equipment and drugs and supplies not always available) | Basic (e.g. unreliable electricity, equipment and drugs and supplies not always available) |
| Access to Upgrading | Eligible to apply for upgrading opportunities after 4 years of service | Eligible to apply for upgrading opportunities after 3 years of service |
| Workload | Heavy workload - You work longer hours because the facility does not have enough staff | Heavy workload - You work longer hours because the facility does not have enough staff |
| Facility Management | The management at the facility is not supportive and makes work more difficult | The management at the facility is supportive and makes work easier |
| Salary | 156,365 MKW per month | 156,365 MKW per month |
| Choice of location | You are randomly assigned to a health facility | You are randomly assigned to a health facility |
| **Which job do you choose?** |  |  |

|  | **Rural Facility** | **Urban Facility** |
| --- | --- | --- |
| Housing | Free Basic housing provided (eg. semi-detached house with two bedrooms) | No housing or housing allowance provided |
| Health Facility Quality | Advanced (e.g. reliable electricity, equipment and drugs and supplies always available) | Basic (e.g. unreliable electricity, equipment and drugs and supplies not always available) |
| Access to Upgrading | Eligible to apply for upgrading opportunities after 3 years of service | Eligible to apply for upgrading opportunities after 3 years of service |
| Workload | Manageable workload - You work within scheduled hours because the facility has sufficient staff | Heavy workload - You work longer hours because the facility does not have enough staff |
| Facility Management | The management at the facility is not supportive and makes work more difficult | The management at the facility is supportive and makes work easier |
| Salary | 125,069 MKW per month | 187,604 MKW per month |
| Choice of location | You are given a choice of district in which you will work | You are given a choice of district in which you will work |
| **Which job do you choose?** |  |  |
