# Supplementary material for: Analysis of policy interventions to attract and retain nurse midwives in rural areas of Malawi: A discrete choice experiment
Source: PLoS One. 2021 Jun 21;16(6):e0253518. doi: 10.1371/journal.pone.0253518 (PMC8216531; doi:10.1371/journal.pone.0253518)
Supplement: Attachment — Submitted filename: Response to Reviewers.docx [file pone.0253518.s005.docx]

**Response to Reviewers**

Thank you for your careful review of our manuscript titled “Analysis of policy interventions to attract and retain nurse midwives in rural areas of Malawi: A discrete choice experiment.” Below please find a response to each point raised by the peer reviewer. The line numbers referenced in our responses below correspond to edits we have made in the “Revised Manuscript with Track Changes” in response to the reviewer comments.

**Points Raised by Reviewer 1**

1. Indicate qualitative data analysis in the method section. In the result section please indicate sex and age of key informant interview result while reporting.

**Response:** We have expanded the qualitative data analysis described in the Methods section starting from line 148. We have also added in sub-headings to clearly demarcate the qualitative and quantitative data analysis sections presented in the Methods. We have added the sex of key informant interviewees in the Results section; however, we did not retain age information for key informant interviews. Focus group discussions were anonymized during transcription, and therefore it is not possible to disaggregate sex and age for individual speakers in the focus groups. Of the four quotations included in the Results, one is from a focus group, and we have clarified this in the manuscript.

2. Please indicate assumptions of conditional logit regression and model fittness test undergone. In the sampling section if you included all study participants please amend sample size calculation area and improve to census type of sampling strategy.

**Response:** We have added the three key assumptions of the model to the DCE data analysis section on line 230 as follows: “The conditional logit model is based on three assumptions: (1) independence of irrelevant alternatives; (2) error terms are independent and identically distributed across observations; and (3) no preference heterogeneity across respondents.” Goodness-of-fit criteria, including Akaike and Bayesian information criteria and pseudo R^2^, were used to assess model fit. These measures are included in the Results section in Table 4. Regarding the sampling strategy, on line 210 we have indicated that to reach our minimum target sample size, we used a census approach to sampling all NMT students and practicing NMTs in a subset of randomly selected NMT training colleges and health facilities.

3. Indicate policy analysis steps used and how you engaged stakeholders in the policy analysis.

**Response:** As elaborated from line 156 onwards, government and other stakeholders were engaged in a workshop to review the qualitative data outputs and prioritize which coded attributes should be included in the DCE questionnaire from a policy perspective, using specific selection criteria. The goal was to ensure that all final attributes included in the DCE questionnaire were interventions that could be practically implemented by the Government of Malawi in the future. As noted in the Discussion section, the final results of this study were then used to inform recommendations in Malawi’s HRH Strategic Plan.
